# *CircARID1A* promotes gastric cancer progression by targeting the *miR-147a*/SNAI2 signaling axis

**DOI:** 10.1080/19336918.2026.2732396

**Published:** 2026-09-30

**Authors:** Kai Guo, Xuemei Yan, Xing Liu, Yu Peng, Weitian Xu

**Affiliations:** a Department of Gastroenterology, General Hospital of Central Theater Command of PLA, Wuhan, Hubei, P. R. China; b Graduate school, The Health and Medical Identification Section of the Wuhan Special Service Center of the Chinese People’s Armed Police Force, Wuhan, Hubei, P. R. China; c Ultrasonography lab, Wuhan University of Science and Technology, Wuhan, Hubei, P. R. China

**Keywords:** Gastric cancer, circ*ARID1A*, *miR-147a*, *SNAI2*

## Abstract

Due to the late stage at which most gastric cancer patients are diagnosed, the prognosis is often poor. Current treatments have limited efficacy for advanced cases. This study delves into the involvement of *circARID1A* and the mechanisms behind its role in gastric cancer progression. GES-1 cell line and GC cell lines HGC-27, AGS, MKN45, and SNU-16 were cultured and subjected to various assays. RNA interference and overexpression techniques were used to modulate *circARID1A* and *miR-147a* levels *in vitro*, while xenograft tumor models in BALB/c nude mice were used to assess tumor growth *in vivo*. Cellular growth, migration, and invasion were assessed using CCK8, colony formation, and Transwell assays. Dual-luciferase reporter assays, qPCR and Western blot were utilized to investigate the interactions between *circARID1A*, *miR-147a*, and *SNAI2*. *CircARID1A* promotes GC cell proliferation, migration, and invasion by regulating the *miR-147a*/SNAI2 axis. *In vivo*, knockdown of *circARID1A* inhibits tumor growth in subcutaneous xenograft models.

## HIGHLIGHTS

- Suppression of *circARID1A* significantly inhibits gastric cancer cell proliferation, migration, and invasion.
- *miR-147a* directly targets and negatively regulates SNAI2 expression, inhibiting its oncogenic effects.
- CircARID1A promotes cell proliferation, migration, and invasion in gastric cancer cells through the *miR-147a/SNAI2* signaling axis.

## GRAPHICAL ABSTRACT

**Figure:**
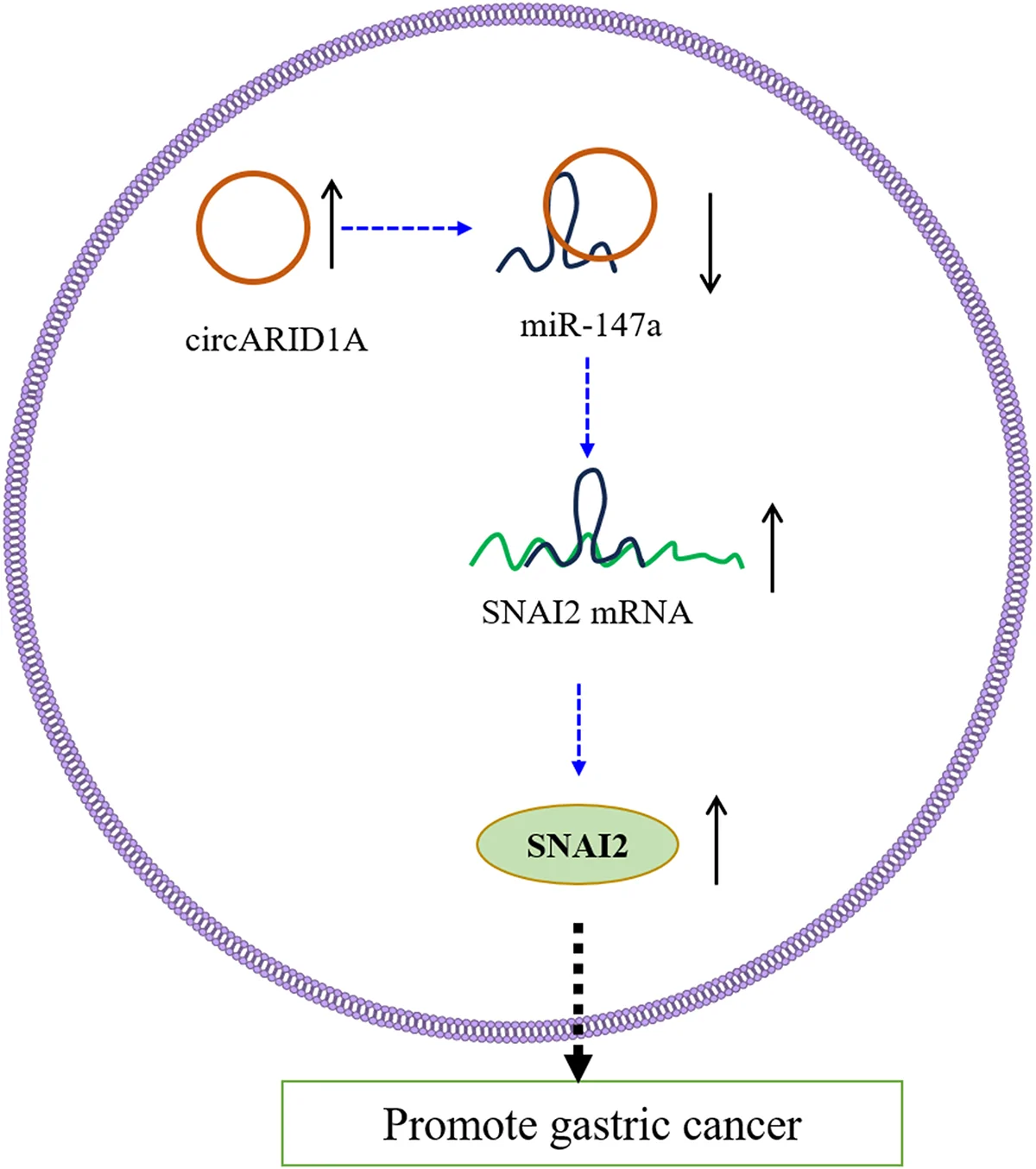
Schematic diagram illustrating the regulatory mechanism of the circARID1A/miR-147a/SNAI2 axis in gastric cancer progression. *CircARID1A* is highly expressed in gastric cancer cells and acts as a miRNA sponge to directly bind and downregulate *miR-147a*. As a direct target of *miR-147a*, *SNAI2* is negatively regulated by *miR-147a* via binding to its 3’UTR. The suppression of *miR-147a* by *circARID1A* leads to elevated *SNAI2* expression, which promotes epithelial-mesenchymal transition (EMT) and enhances the proliferation, migration, and invasion of gastric cancer cells, thereby facilitating tumor progression. In brief, *circARID1A* drives gastric cancer development through the *miR-147a*/SNAI2 signaling axis, providing a potential therapeutic target for gastric cancer.

## Introduction

Globally, gastric cancer (GC) is recognized as the fifth most prevalent form of cancer and the third most common cause of death due to cancer [1,2]. The absence of distinct symptoms in the early stages often results in late-stage diagnoses, leading to a poor prognosis [3,4]. The standard treatment approaches for GC currently consist of chemotherapy, radiotherapy, surgery, and targeted therapy; however, these treatments have limited effectiveness for advanced patients and are often associated with high recurrence rates and low survival rates [5,6]. Hence, delving into the molecular pathways of GC and finding novel therapeutic targets is vital for better patient prognosis.

Non-coding RNAs have recently been recognized for their important role in the onset and development of cancer. Circular RNAs (circRNAs), a newly identified class of non-coding RNAs, are distinguished by their unique circular structure, which grants them enhanced stability and specificity within cells [7,8]. CircRNAs regulate gene expression by interacting with microRNAs (miRNAs) or directly binding to proteins, thereby affecting various biological functions of cells [9–12]. In GC, several circRNAs have been found to regulate cellular migration, proliferation and invasion [13–16]. Existing studies have only preliminarily indicated that it may participate in cancer progression by regulating downstream molecules. For instance, Ma et al [17] demonstrated that *circARID1A* binds to IGF2BP3 in GC and promotes cancer growth through the assembly of a *circARID1A*-IGF2BP3-SLC7A5 RNA-protein complex. Consistent with this notion, emerging single-cell analyses indicate that aberrant gene expression in gastric cancer is closely associated with malignant progression and remodeling of the tumor immune microenvironment [18,19]. However, this remains the only study currently available on *circARID1A* in gastric cancer, and the detailed molecular mechanisms mediated by *circARID1A* require further investigation.

These short non-coding RNAs, known as miRNAs, are about 22 nucleotides in length and primarily modulate gene expression by attaching to the 3’ untranslated regions (3’UTR) of their target mRNAs, thereby inhibiting mRNA translation or promoting its degradation [20,21]. *MiR-147a* has been shown to exert tumor-suppressive effects across multiple types of cancer, including neuroblastoma, lung cancer, ovarian cancer, colorectal cancer, and melanoma [22,23]. However, there are no reports on *miR-147a* in GC. The starBase database predicts that *miR-147a* has target binding sites with *circARID1A*, suggesting that *miR-147a* might be crucial in the advancement of GC.

*SNAI2*, a member of the Snail Family of transcriptional repressors, is frequently upregulated in various cancers and plays a crucial role in driving cancer cell migration and invasion [24,25]. The upregulation of *SNAI2* activates the Snail/Slug pathway, promoting the proliferation, metastasis, and invasion of in GC cells [26–28]. However, research on *SNAI2* in GC is still insufficient, and its upstream and downstream regulatory mechanisms require further investigation. The starBase database predicts that *SNAI2* has binding sites with *miR-147a*, further supporting the hypothesis that *miR-147a* may regulate *SNAI2* to exert its effects in GC.

This study seeks to investigate the mechanism through which circARID1A promotes malignant proliferation, invasion and migration in GC cells by targeting the *miR-147a*/SNAI2 signaling axis. By elucidating the specific mechanisms of this regulatory axis in GC, this study hopes to identify new potential targets for the diagnosis and therapy of GC, with significant clinical implications.

## Methods

### Xenograft tumor model in nude mice

Female BALB/c nude mice (4–6 weeks old, 18–20 g) were purchased from the Experimental Animal Center, and all experiments were approved by the Institutional Animal Care and Use Committee of General Hospital of Central Theater Command of PLA. Mice were housed in a specific pathogen-free (SPF) environment at a controlled temperature of 22–24°C, relative humidity of 50–60%, and a 12-hour light/dark cycle, with free access to food and water. After a one-week acclimatization period, AGS gastric cancer cells with different treatments (1 × 10^6^ cells in 100 µL PBS) were injected subcutaneously into the right flank of each mouse, with treatment groups including sh-NC, sh-circARID1A, mimics NC, *miR-147a* mimics, *miR-147a* mimics+oe-NC, and *miR-147a* mimics+oe-*SNAI2*, with 5 mice per group. Tumor length and width were measured every three days using a caliper, and tumor volume was calculated as V = (length × width [2])/2. After four weeks, mice were euthanized by intraperitoneal injection of an overdose of sodium pentobarbital (150 mg/kg; Sigma-Aldrich #No. P3761), And, tumors were excised, weighed, and photographed.

### Immunohistochemistry

Tumor tissues were fixed in 10% neutral-buffered formalin (Sigma-Aldrich, USA, #HT501128) for 24 hours, then dehydrated, paraffin-embedded, and sectioned at a thickness of 4 µm. Sections were deparaffinized in xylene (Sigma-Aldrich, USA, #214736), followed by rehydration through a graded ethyl alcohol (Sigma-Aldrich, USA, #E7023) series (100%, 95%, and 75%). Antigen retrieval was performed by heating the sections in citrate buffer (pH 6.0; Sigma-Aldrich, USA, #C2488-100 ML) to boiling in a microwave and maintaining for 10 minutes. Sections were then blocked with 5% goat serum (Beyotime, China, #C0265) in PBS at room temperature for 30 minutes to reduce nonspecific binding. Ki67 primary antibody (1:200 dilution, Abcam, UK, #ab15580) was applied to the sections and incubated overnight at 4°C. The following day, sections were rinsed with PBS and incubated with biotinylated secondary antibody (1:200 dilution, Beyotime, China, #A0277) at room temperature for 1 hour. Detection was conducted using DAB substrate solution (Beyotime, China, #P0203), with a development time of approximately 5 minutes, followed by rinsing in tap water to stop the reaction. Sections were counterstained with hematoxylin, dehydrated through an alcohol gradient, and mounted.

### Cell culture

The GES-1 human gastric epithelial cell line and GC cell lines HGC-27, AGS, MKN45, and SNU-16 were obtained from the Cell Bank of the Chinese Academy of Sciences. Cells were cultured in Dulbecco’s Modified Eagle Medium (DMEM, Gibco, USA, #11965092) or RPMI-1640 medium (Gibco, USA, # 11,875,093), both supplemented with 10% fetal bovine serum (FBS, Gibco, USA, #16000044) and 1% penicillin-streptomycin (Gibco, USA, #15140122), at 37°C in a 5% CO_2_ atmosphere. Cells were sub-cultured every 3–4 days to ensure they were in the logarithmic growth phase for experiments.

### Cell transfection

After being grown in 6-well plates to 70–80% confluency, the cells were transfected using Lipofectamine 3000 reagent (Invitrogen, USA, #L3000015). For *circARID1A* knockdown experiments, shRNA expression vectors (sh-*circARID1A*) were used, with shRNA negative control (sh-NC) as the control. For overexpression experiments, circARID1A expression vectors (oe-*circARID1A*) were used, with empty vectors (oe-NC) as the control. Additionally, *miR-147a* and *SNAI2* transfection vectors were used in relevant experiments, including *miR-147a* mimics, oe-*SNAI2* and sh-*SNAI2*. All vectors were sourced from GenePharma. Cells were harvested 48 hours post-transfection for subsequent experiments. Experiments were performed in both AGS and HGC-27 cell lines, with identical transfection procedures applied to both cell lines.

### Cell viability assay

The CCK8 kit (Dojindo, Japan, #CK04-01) was used to measure cell viability. Transfected cells were distributed into 96-well plates at a concentration of 5000 cells per well, with five replicate wells per group. After 48 hours, 10 μL of CCK8 solution was introduced into each well, followed by a 2-hour incubation at 37°C. Cell viability was then evaluated by measuring the optical density (OD) at 450 nm using a microplate reader.

### Colony formation assay

Transfected cells were plated into 6-well plates at a density of 1000 cells per well, with three replicate wells per group. After culturing the cells for 10–14 days, visible colonies were observed. The cells were subsequently washed twice with PBS, fixed with methanol (Sinopharm Chemical Reagent, China, #SCRC CSDS67‑56‑1) for 10–15 minutes, and stained with 0.1% crystal violet (Solarbio, China, # G1063) for 20 minutes. The excess stain was rinsed off with tap water, and the colonies were photographed and counted under a microscope.

### *Cell migration and* in vitro *invasion assays*

Using Transwell chambers with 8 μm pores (Corning, USA, # 3428), transfected cells were resuspended in serum-free medium and introduced into the upper chambers at a density of 1 × 10^5^ cells/200 μL (AGS and HGC-27 cell lines). Medium with 10% FBS was added to the lower chambers as a chemoattractant. After incubating for 24 hours at 37°C, the cells were fixed with methanol and stained with 0.1% crystal violet. Cells that did not migrate were carefully removed with a cotton swab, and those that migrated to the underside of the membrane were counted under a microscope.

The procedure for this assay was almost identical to the migration assay, except that Matrigel (diluted 1:8, BD Biosciences, USA, #356234) was used to pre-coat the upper chambers. All other steps mirrored those of the migration assay.

### Dual-luciferase reporter assay

Wild-type (WT) and mutant (MUT) 3’UTR fragments of *circARID1* A and *SNAI2* were cloned into luciferase reporter vectors (Promega, USA, #E1751). These constructs were co-transfected with *miR-147a* mimics (GenePharma, China, #B02001) or negative control (NC, GenePharma, China, # B04002) into AGS cells using Lipofectamine 3000 (Invitrogen, USA, # L3000015). After 48 hours, the Dual-Luciferase Reporter Assay System (Promega, USA, # E1910) was used to measure luciferase activity, with firefly luciferase activity being normalized against *Renilla luciferase* (Promega, USA, #E2241) activity.

### Quantitative PCR (qPCR)

Using TRIzol reagent (Invitrogen, USA, # 15,596,026), total RNA was isolated and then reverse-transcribed into cDNA according to the PrimeScript RT reagent Kit (Takara, Japan, #RR037A). qPCR was conducted on an ABI 7500 real-time PCR system with the SYBR Green PCR Kit (Takara, Japan, #RR820A). The thermal cycling protocol included an initial denaturation at 95°C for 30 seconds, followed by 40 cycles of 95°C for 5 seconds, and annealing/extension at 60°C for 20 seconds. Primers were synthesized by Sangon Biotech (Shanghai, China), with the following sequences: *circARID1A* forward primer: 5’-GCCGAGGAGAAGAAGGTGTT-3,“ reverse primer: 5”-GTTTCCCATTTTGTTGTTGGTTT-3“; *miR-147a* forward primer: 5”-GCGGAAGACGTTTTGAACTG-3,“ reverse primer: 5”-GTGCAGGGTCCGAGGT-3“; *SNAI2* forward primer: 5”-AGATGCATATTCGGACCCAC-3,“ reverse primer: 5”-TGTTGCAGTGAGGGCAAGAG-3“; *GAPDH* forward primer: 5”-GAAGGTGAAGGTCGGAGTC-3,“ reverse primer: 5”-GAAGATGGTGATGGGATTTC-3“; U6 forward primer: 5”-CTCGCTTCGGCAGCACA-3,“ reverse primer: 5”-AACGCTTCACGAATTTGCGT-3.’ Relative quantification was performed using the 2^^−ΔΔCt^ method.

### Western blot

Total protein was isolated from cells or tumor tissues using RIPA buffer (Beyotime, China, #P0013B). The extracted proteins were then separated via SDS-PAGE and transferred to PVDF membranes (Millipore, USA, #IPVH00010). The membranes were incubated with the following primary antibodies: anti-SNAI2 (Abcam, UK, ab27568), anti-N-cadherin (Cell Signaling Technology, USA, #13116), anti-E-cadherin (Cell Signaling Technology, USA, #3195), anti-Snail (Cell Signaling Technology, USA, #3879), anti-Vimentin (Cell Signaling Technology, USA, #5741), and anti-*GAPDH* (Cell Signaling Technology, USA, #5174). Following this, the membranes were incubated for 1 hour with HRP-conjugated secondary antibodies (anti-mouse IgG, Cell Signaling Technology, USA, #7076; anti-rabbit IgG, Cell Signaling Technology, USA, #7074). Bands were detected using ECL detection reagent (Millipore, USA, #WBKLS0500) and analyzed with ImageJ software. Western blot analyses for rescue experiments were conducted in both AGS and HGC-27 cells.

### Rnase R digestion stability assay

Total RNA was extracted from AGS cells and divided into two groups: one group was treated with RNase R (Epicenter, USA, # RNR07250) at a final concentration of 3 U/μg, and the other group was left untreated as a control. Both groups were incubated at 37°C for 30 minutes. Subsequently, RNA was re-extracted using TRIzol reagent, and reverse transcription into cDNA was performed according to the instructions of the PrimeScript RT reagent Kit. Quantitative PCR (qPCR) was used to detect the expression levels of *circARID1A* and linear *ARID1A*, with *GAPDH* as the internal reference gene. The relative expression levels were calculated using the 2^^-ΔΔCt^ method to evaluate the resistance of *circARID1A* to RNase R degradation.

### Nucleocytoplasmic separation assay

AGS cells were subjected to nucleocytoplasmic separation using a Nucleocytoplasmic Separation Kit (Beyotime, China, #P0028) following the manufacturer’s instructions. Specifically, cells were collected and lysed, and the cytoplasmic fraction was obtained by centrifugation; subsequently, the nuclear pellet was lysed to obtain the nuclear fraction. qPCR was used to detect the expression level of *circARID1A* in both cytoplasmic and nuclear fractions, with U6 as the nuclear internal reference gene and *GAPDH* as the cytoplasmic internal reference gene. The relative distribution ratio of *circARID1A* in the nucleus and cytoplasm was calculated.

### RNA fluorescence in situ hybridization (RNA-FISH)

Experiments were performed according to the instructions of the RNA-FISH Detection Kit (RiboBio, China, #C10922). First, AGS cells were seeded into confocal culture dishes and cultured until they adhered to the dishes. After fixing and permeabilizing the cells, FAM-labeled *circARID1A*-specific probes (Genepharma, China, #GP-FAM-CA001) were added and incubated overnight at 37°C. The cells were then washed with washing buffer to remove unbound probes, and DAPI staining solution was added to stain the cell nuclei for 5 minutes. Finally, the subcellular localization of *circARID1A* was observed using a laser confocal microscope (Zeiss LSM 780, Zeiss, Germany), and images were captured.

### Statistical analysis

Experimental data were analyzed using SPSS 29.0 software and expressed as mean ± standard deviation (mean ± SD). Comparisons between groups were performed using Student’s t-test, with *p* < .05 considered statistically significant.

## Results

### *Levels of* circARID1A *and* miR-147a *expression in GC cells, and characterization of* circARID1A

The results showed that compared to GES-1, all GC cell lines (AGS, HGC-27, MKN45, SNU-16) exhibited significantly increased expression of *circARID1A* and significantly decreased expression of *miR-147a*, with the highest *circARID1A* expression in the AGS cell line (Figure 1(A,B)). To verify the circular nature and molecular characteristics of *circARID1A*, we performed RNase R digestion assay, nucleocytoplasmic separation assay, RNA-FISH assay, and Sanger sequencing. RNase R digestion results showed that the expression level of linear ARID1A was significantly reduced after RNase R treatment, while *circARID1A* expression remained almost unchanged, indicating its resistance to RNase R degradation (Figure 1(C)). Nucleocytoplasmic separation assay revealed that *circARID1A* was predominantly localized in the cytoplasm, with cytoplasmic expression accounting for over 75% (Figure 1(D)). RNA-FISH further confirmed that *circARID1A* fluorescent signals were mainly concentrated in the cytoplasm, with no obvious overlap with DAPI-labeled nuclei (Figure 1(E)).

**Figure 1.**
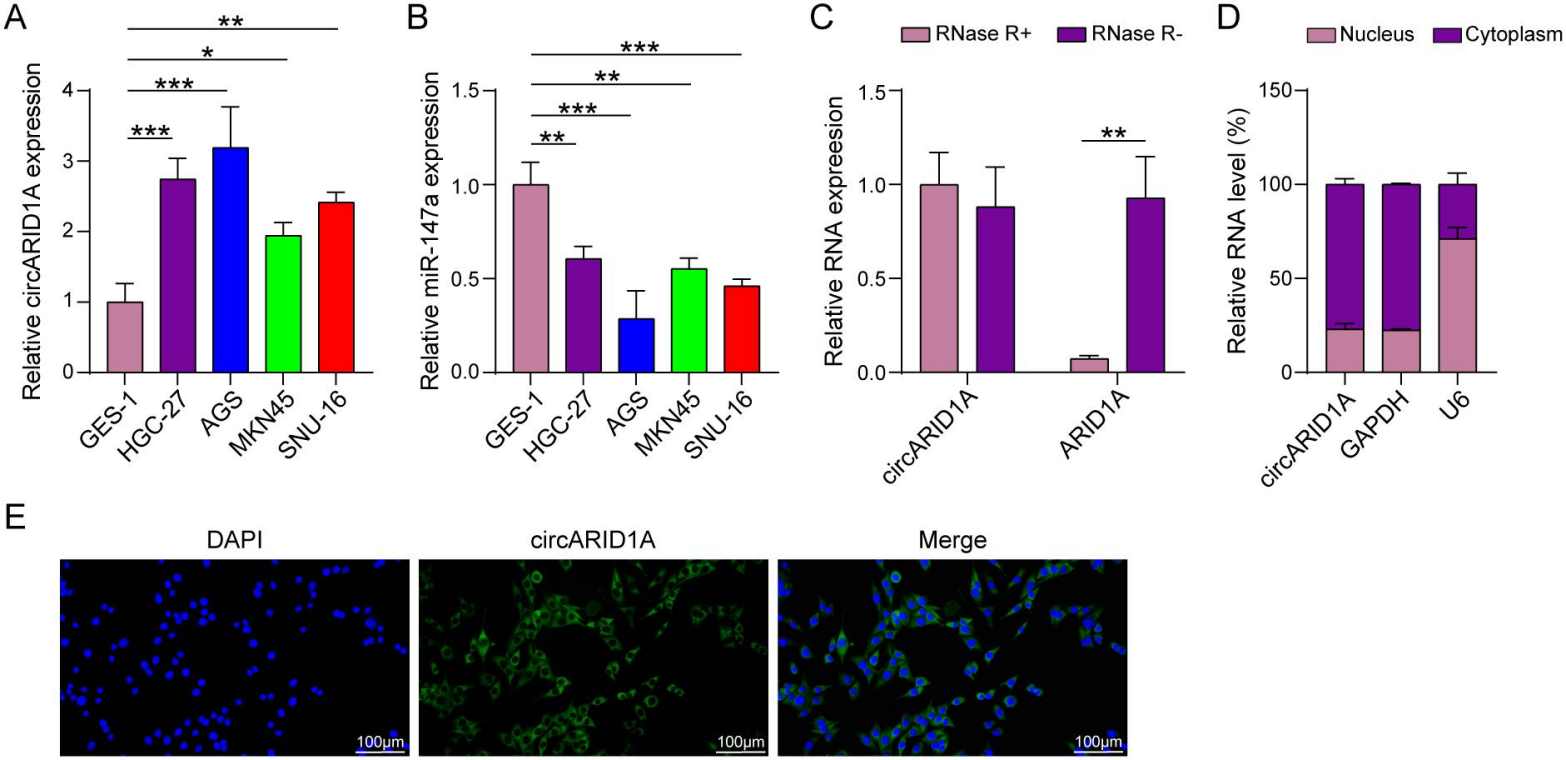
Expression levels of *circARID1A* and *miR-147a* in GC cells. Cultured cell lines including HGC-27, AGS, MKN45, and SNU-16. (A)*circARID1A* expression was detected by qPCR. (B) qPCR analysis of *miR-147a* expression in normal gastric epithelial cell line (GES-1) and GC cell lines (HGC-27, AGS, MKN45, SNU-16). (C) qPCR detection of *circARID1A* and *ARID1A* expression after RNase R treatment. (D) qPCR analysis of *circARID1A* expression in nucleus and cytoplasm after nucleocytoplasmic separation. (E) RNA-FISH assay for *circARID1A* localization in AGS cells (scale bar = 100 μm). *n* = 3, *p < .05, **p < .01, ***p < .001.

### *Knockdown of* circARID1A *inhibits malignant proliferation of GC cells*

The results showed that sh-*circARID1A*#1, sh-*circARID1A*#2, and sh-*circARID1A*#3 all significantly reduced the expression of *circARID1A*, with sh-*circARID1A*#2 showing the most significant knockdown effect (Figure 2(A)). Knockdown of *circARID1A* significantly reduced the cell viability of AGS (Figure 2(B)) and inhibited the colony-forming ability of in AGS cells (Figure 2(C)). Additionally, knockdown of *circARID1A* significantly reduced the migration and *in vitro* invasion abilities of GC cells (Figure 2(D)). After knocking down *circARID1A*, E-cadherin protein expression was markedly elevated, whereas the levels of Vimentin, N-cadherin, and Snail were notably reduced (Figure 2(E)). These findings indicate that *circARID1A* might influence the invasive potential of GC cells by modulating the expression of genes related to EMT.

**Figure 2.**
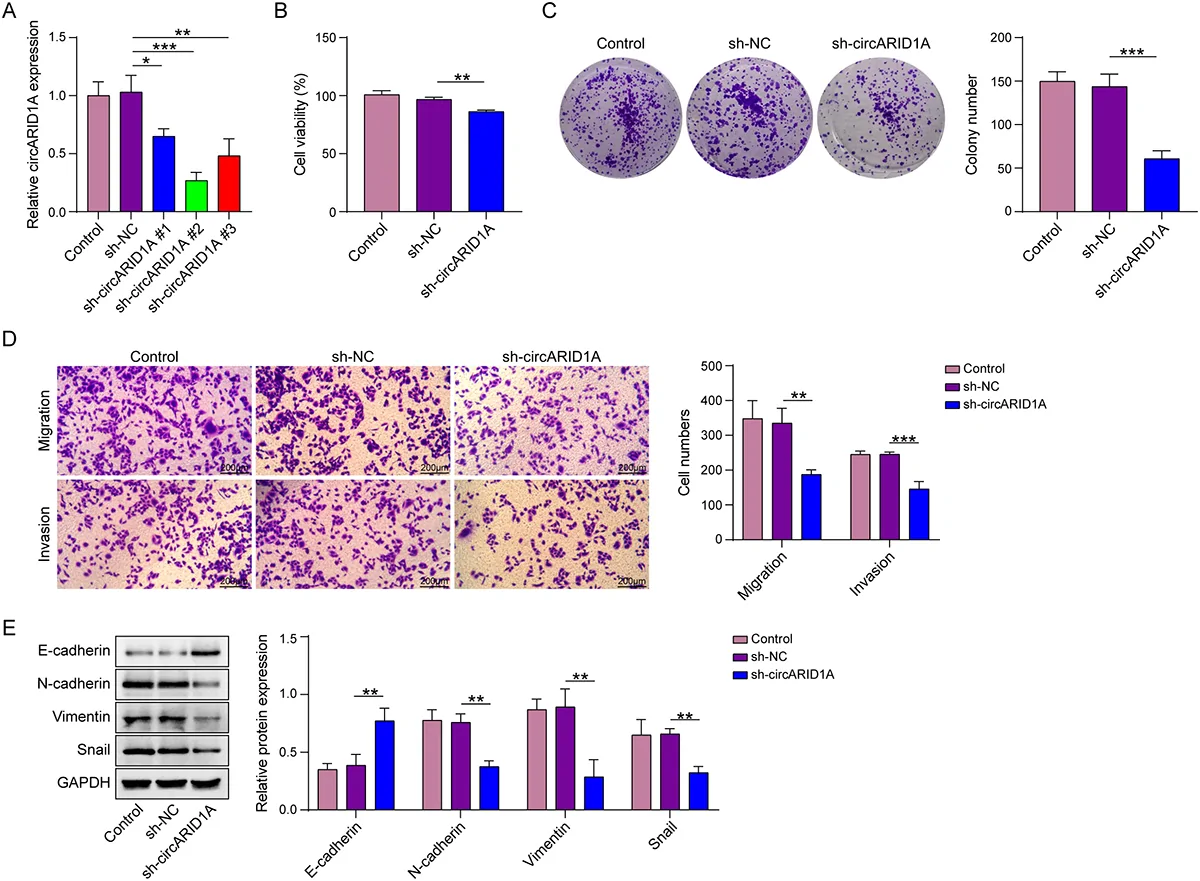
Knockdown of *circARID1A* inhibits malignant proliferation of GC cells. (A) qPCR analysis of *circARID1A* expression after transfection with different shRnas. (B) CCK8 assay to evaluate the effect of *circARID1A* knockdown on GC cell viability. (C) Colony formation assay to evaluate the effect of *circARID1A* knockdown on cell proliferation. (D) Transwell assay to evaluate the effect of *circARID1A* knockdown on cell migration and invasion. (E) Western blot analysis of EMT-related protein expression after *circARID1A* knockdown. Data are presented as mean ± standard deviation. *n* = 3, *p < .05, **p < .01, ***p < .001.

### *Knockdown of* circARID1A *inhibits GC tumor growth in nude mouse subcutaneous xenograft models*

*In vivo* experiments using BALB/c nude mice showed that knockdown of *circARID1A* significantly suppressed gastric cancer progression. Compared to the sh-NC group, *circARID1A* expression was significantly downregulated in the sh-*circARID1A* group (Figure 3(A)). Tumor imaging and tumor volume measurements revealed that tumors in the sh-*circARID1A* group were visibly smaller, with significantly reduced tumor volumes compared to the sh-NC group (Figure 3(B,C)). Tumor weights were also significantly lower in the sh-*circARID1A* group (Figure 3(D)). Immunohistochemical analysis of Ki67 demonstrated a significant reduction in Ki67-positive cells in the sh-*circARID1A* group, indicating reduced proliferative activity in the tumor tissues (Figure 3(E)). These results suggest that knockdown of *circARID1A* inhibits tumor growth in gastric cancer.

**Figure 3.**
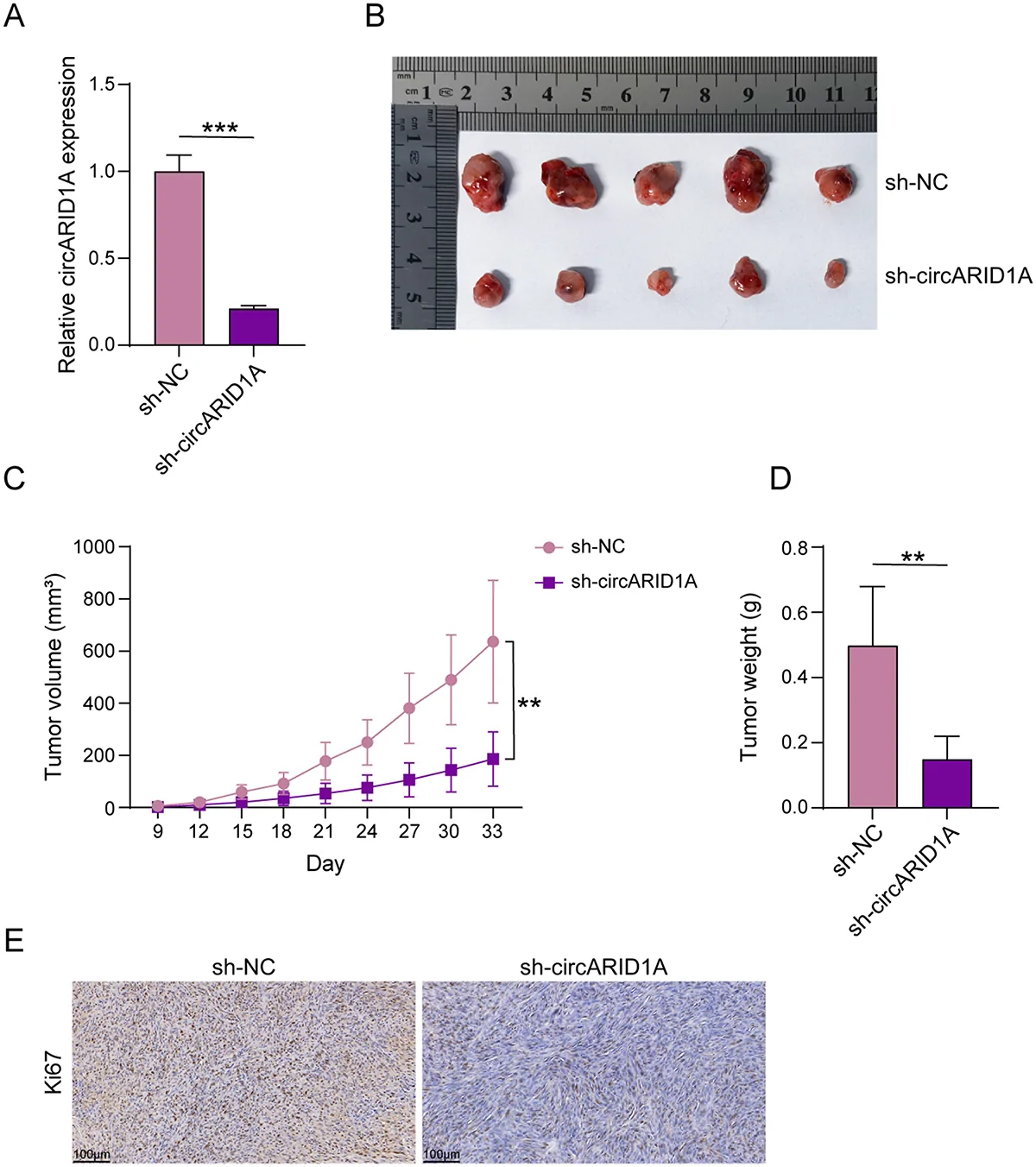
Knockdown of *circARID1A* inhibits GC tumor growth in nude mice. (A) qPCR analysis of *circARID1A* expression in tumor tissues after *circARID1A* knockdown. (B) Representative images of tumors from sh-NC and sh- *circARID1A* groups. (C) Tumor volume growth curve in nude mice injected with AGS cells transfected with sh-NC or sh- *circARID1A*. (D) Tumor weight in the sh-NC and sh- *circARID1A* groups. (E) Immunohistochemical staining of Ki67 to assess cell proliferation in tumor tissues from sh-NC and sh- *circARID1A* groups. Data are presented as mean ± standard deviation. *n* = 5, **p < .01, ***p < .001.

### CircARID1A *targets miR-147a to regulate its expression*

*CircARID1A* was predicted to have potential binding sites for *miR-147a* via starBase database (Figure 4(A)). It showed that *miR-147a* mimics significantly reduced the luciferase activity of WT *circARID1A* 3’UTR fragments, but had no significant effect on MUT type (Figure 4(B)), indicating that *circARID1A* can directly bind and negatively regulate *miR-147a* expression. Knockdown of *circARID1A* significantly reduced its own expression, while overexpression of *circARID1A* promoted its own expression (Figure 4(C)). Additionally, knockdown of *circARID1A* significantly upregulated *miR-147a* expression, whereas overexpression of *circARID1A* significantly downregulated *miR-147a* expression (Figure 4(D)). These findings further validate that *circARID1A* suppresses *miR-147a* expression through direct binding.

**Figure 4.**
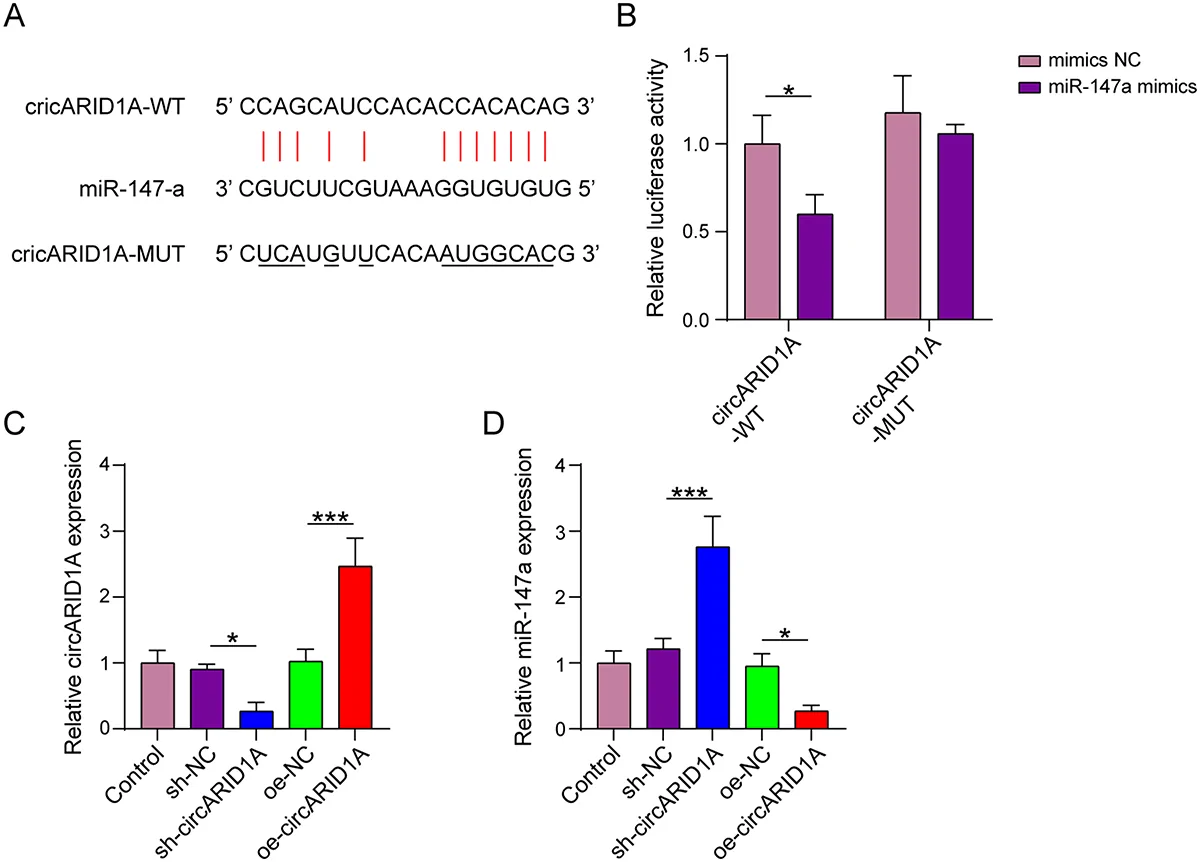
*CircARID1A* targets and regulates *miR-147a* expression. (A) starBase database prediction of binding sites between *circARID1A* and *miR-147a*. (B) dual-luciferase reporter assay to validate the interaction between *circARID1A* and *miR-147a*. (C) qPCR analysis of circARID1A expression after *circARID1A* knockdown and overexpression. (D) qPCR analysis of *miR-147a* expression after *circARID1A* knockdown and overexpression. Data are presented as mean ± standard deviation. *n* = 3, *p < .05, ***p < .001.

### SNAI2 *is a target gene of* miR-147a

The starBase database predicted that the 3’UTR region of *SNAI2* has potential binding sites for *miR-147a* (Figure 5(A)). *MiR-147a* mimics caused a significant drop luciferase activity for the WT SNAI2 3’UTR fragment, but did not alter the activity in the MUT type (Figure 5(B)), indicating that *miR-147a* can directly bind and negatively regulate *SNAI2* expression. *MiR-147a* mimics significantly elevated the expression level of *miR-147a*, while *miR-147a* inhibitors significantly decreased its expression level (Figure 5(C)). Furthermore, *miR-147a* mimics significantly lowered the mRNA and protein expression levels of *SNAI2*, while *miR-147a* inhibitors substantially upregulated *SNAI2* expression (Figure 5(D, E)). These findings further validate that *miR-147a* suppresses *SNAI2* expression by binding directly to the 3’UTR region of *SNAI2*.

**Figure 5.**
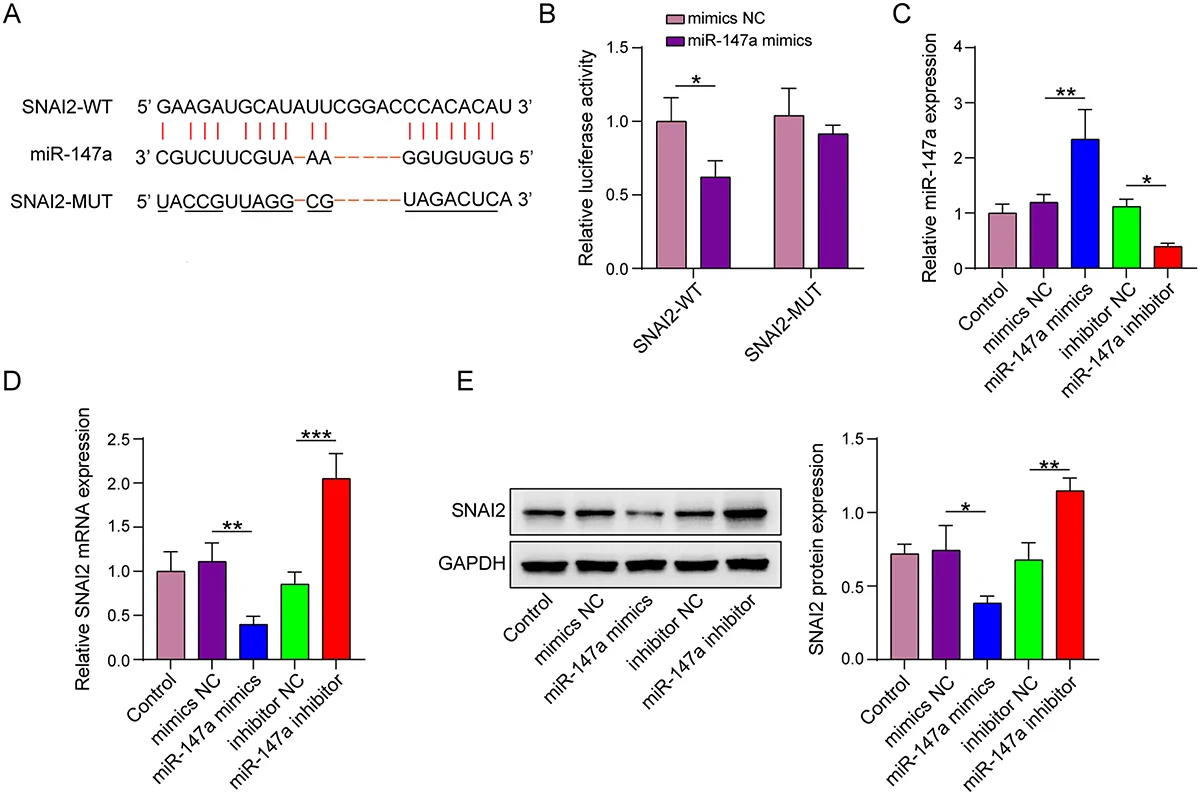
*SNAI2* is a target gene of *miR-147a*. (A) starBase database prediction of binding sites between *miR-147a* and *SNAI2*, along with mutation analysis. (B) Dual-luciferase reporter assay to validate the interaction between *miR-147a* and *SNAI2*. (C) qPCR analysis of *miR-147a* expression after transfection with *miR-147a* mimics and *miR-147a* inhibitor. (D) qPCR analysis of *Snai2* mRNA expression after transfection with *miR-147a* mimics and *miR-147a* inhibitor. (E) Western blot analysis of SNAI2 protein expression after transfection with *miR-147a* mimics and *miR-147a* inhibitor. Data are presented as mean ± standard deviation. *n = 3*, *p < .05, **p < .01, ***p < .001.

### MiR-147a *targets SNAI2 to inhibit malignant proliferation of GC cells*

*MiR-147a* mimics significantly upregulated *miR-147a* expression while downregulating *SNAI2* mRNA levels, and co-transfection with oe-*SNAI2* partially restored *SNAI2* expression (Figure 6(A)). While *miR-147a* mimics led to a reduction in SNAI2 protein expression, co-transfection with the *SNAI2* overexpression vector reinstated SNAI2 levels (Figure 6(B)). *MiR-147a* mimics significantly reduced the viability, colony-forming ability, migration, and *in vitro* invasion in AGS cells, while *SNAI2* overexpression partially restored these abilities (Figure 6(C–E)). Additionally, *miR-147a* mimics significantly increased E-cadherin protein expression while significantly decreasing the protein levels of Vimentin, N-cadherin, and Snail, and co-transfection with the *SNAI2* overexpression vector partially reversed these changes (Figure 6(F)). It indicated that *miR-147a* exerts its tumor-suppressive effects in GC cells primarily through the downregulation of *SNAI2*.

**Figure 6.**
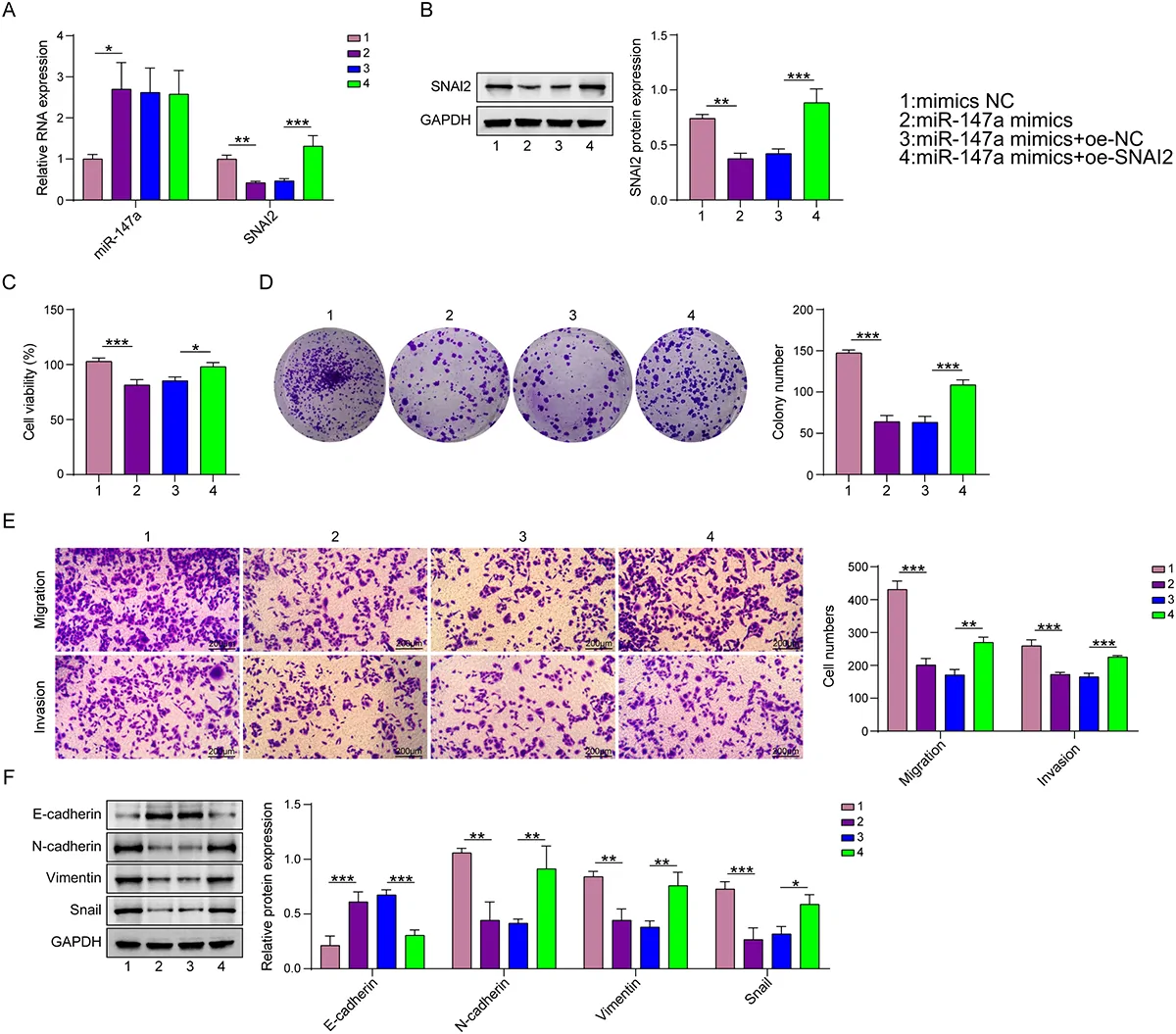
*miR-147a* targets *SNAI2* to inhibit malignant proliferation of GC cells. (A) qPCR analysis of *miR-147a* and *SNAI2* mRNA expression. (B) Western blot analysis of SNAI2 expression. (C) CCK8 assay to evaluate the effect of *miR-147a* mimics and *SNAI2* on GC cell viability. (D) colony formation assay to evaluate the effect of *miR-147a* mimics and *SNAI2* on cell proliferation. (E) Transwell assay to evaluate the effect of *miR-147a* mimics and *SNAI2* on GC cell migration and invasion. (F) Western blot analysis of E-cadherin, Vimentin, N-cadherin, and Snail protein expression. Data are presented as mean ± standard deviation. *n* = 3, *p < .05, **p < .01, ***p < .001.

### miR-147a *targets SNAI2 to suppress GC tumor growth in nude mice*

*MiR-147a* was found to target *SNAI2* and suppress gastric cancer progression *in vivo*. qPCR analysis demonstrated that *miR-147a* mimics significantly upregulated *miR-147a* expression while downregulating *SNAI2* expression in tumors. Overexpression of *SNAI2* partially restored *SNAI2* expression but had no effect on *miR-147a* levels (Figure 7(A)). Western blot analysis showed that *miR-147a* mimics significantly reduced SNAI2 protein expression, while *SNAI2* overexpression reversed this effect (Figure 7(B)). Tumor size was significantly reduced in the *miR-147a* mimics group compared to the mimics NC group, indicating that *miR-147a* suppressed tumor growth. *SNAI2* overexpression partially restored tumor growth (Figure 7(C,D)). Tumor weights were significantly lower in the *miR-147a* mimics group and partially restored in the *miR-147a* mimics+oe-*SNAI2* group (Figure 7(E)). *miR-147a* mimics decreased Ki67-positive cells in tumor tissues, suggesting reduced proliferation, while *SNAI2* overexpression partially reversed this effect (Figure 7(F)). These results indicate that *miR-147a* inhibits gastric cancer tumor growth by targeting *SNAI2*.

**Figure 7.**
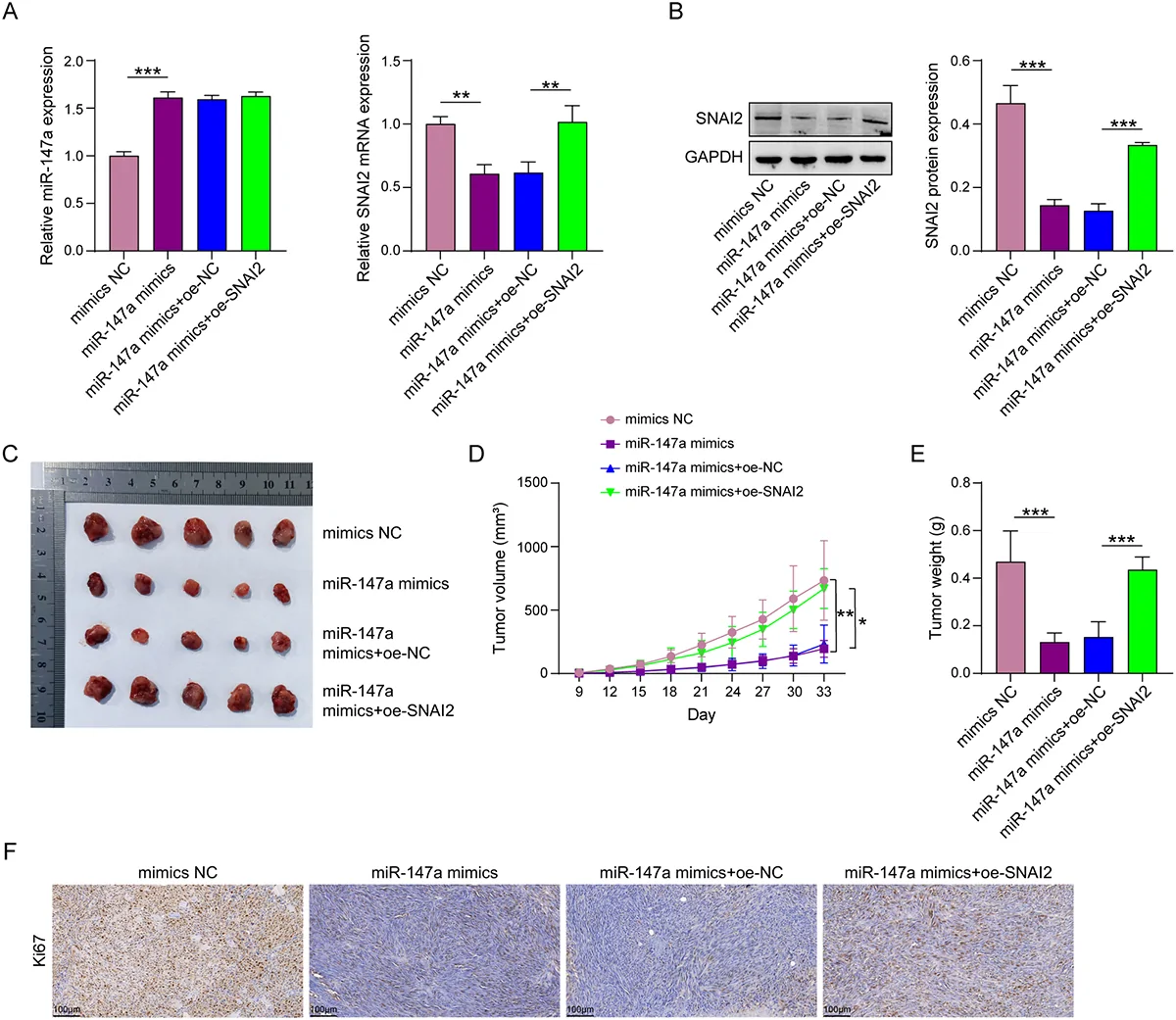
*miR-147a* targets *SNAI2* to suppress GC tumor growth in nude mice. (A) qPCR analysis of *miR-147a* and SNAI2 mRNA expression in tumor tissues after transfection with *miR-147a* mimics or co-transfection with *miR-147a* mimics and SNAI2 overexpression (oe-SNAI2). (B) Western blot analysis of SNAI2 protein expression. (C) Representative images of tumors from mimics NC, *miR-147a* mimics, *miR-147a* mimics+oe-NC, and *miR-147a* mimics+oe- *SNAI2* groups. (D) Tumor volume growth curve in nude mice from the four treatment groups. (E) Tumor weight in the four groups. (F) immunohistochemical staining of Ki67 to assess cell proliferation in tumor tissues from the four groups. Data are presented as mean ± standard deviation. *n* = 5, *p < .05, **p < .01, ***p < .001.

### CircARID1A *promotes malignant proliferation of GC cells by targeting the miR-147a/SNAI2 signaling axis*

Knockdown of *circARID1A* significantly reduced its own expression, upregulated *miR-147a* expression, and downregulated *SNAI2* expression; transfection with *miR-147a* inhibitor did not affect *circARID1A* expression but downregulated *miR-147a* and upregulated *SNAI2*; knockdown of *SNAI2* did not alter *circARID1A* or *miR-147a* levels but reduced *SNAI2* expression (Figure 8(A)). Knockdown of *circARID1A* significantly reduced SNAI2 protein expression, *miR-147a* inhibitor increased it, and knockdown of *SNAI2* reduced it again (Figure 8(B)). Knockdown of *circARID1A* significantly reduced GC cell viability (Figure 8(C)) and colony formation ability (Figure 8(D)), and inhibited cell migration and *in vitro* invasion (Figure 8(E)); *miR-147a* inhibitor significantly offset the effects of sh-*circARID1A*, while simultaneous knockdown of *SNAI2* reversed the function of *miR-147a* inhibitor. Consistent results were obtained in HGC-27 cells, confirming the findings are not cell-line specific. Silencing *circARID1A* notably increased E-cadherin expression while decreasing the levels of Vimentin, N-cadherin, and Snail; in contrast, *miR-147a* inhibitor reversed this trend, and knockdown of *SNAI2* further upregulated E-cadherin and downregulated other EMT-related proteins (Figure 8(F)). Consistent results were obtained in HGC-27 cells, confirming the findings are not cell-line specific (**Fig S1**). These results underscored that *circARID1A* promotes gastric cancer proliferation, migration, and *in vitro* invasion through the *miR-147a*/SNAI2 axis, and its knockdown inhibits tumor cell growth.

**Figure 8.**
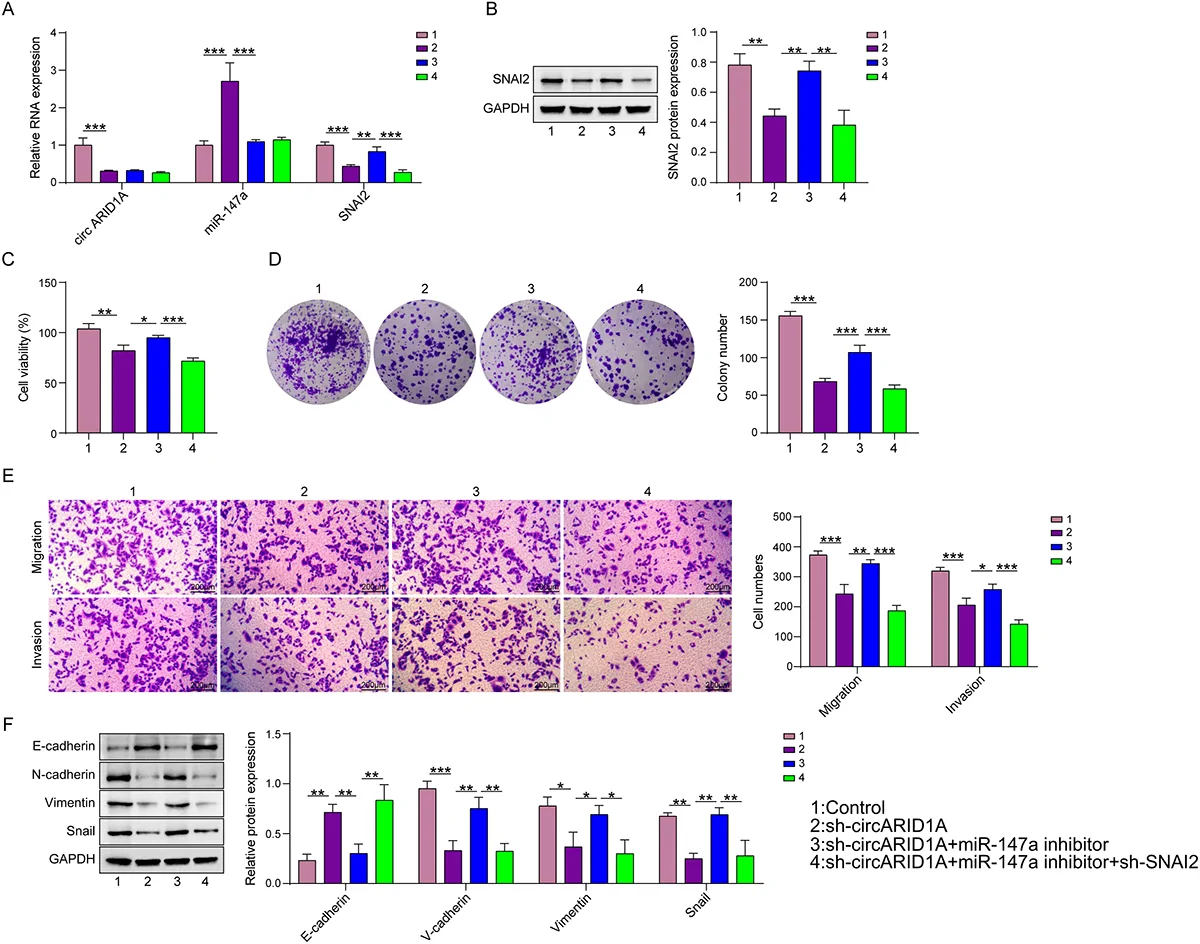
*CircARID1A* promotes malignant proliferation of GC cells by targeting the *miR-147a* /SNAI2 signaling axis. (A) qPCR analysis of *circARID1A,miR-147a*, and *SNAI2* mRNA expression. (B) Western blot analysis of SNAI2 protein expression. (C) CCK8 assay to evaluate cell viability. (D) Colony formation assay to evaluate cell proliferation ability. (E) Transwell assay to evaluate cell migration and invasion abilities. (F) Western blot analysis of E-cadherin, Vimentin, N-cadherin, and Snail protein expression. Data are presented as mean ± standard deviation. *n* = 3, *p < .05, **p < .01, ***p < .001.

## Discussion

This study focuses on the mechanisms involving *circARID1A*, *miR-147a*, and *SNAI2* in GC cells, revealing that *circARID1A* enhances malignant proliferation of GC both *in vitro* and *in vivo* through the *miR-147a*/SNAI2 signaling axis. The results indicate that *circARID1A* is abundantly expressed in GC, and silencing of *circARID1A* inhibits cellular growth, migration and invasion. *In vivo* experiments demonstrated that *circARID1A* knockdown significantly inhibited tumor growth. Further molecular mechanism studies show that *circARID1A* negatively regulates *miR-147a*, thereby upregulating its target gene *SNAI2*, ultimately promoting the malignant behavior of GC cells.

The subcellular localization of circRNAs dictates their functions: cytoplasmic circRNAs often act as miRNA sponges, while nuclear circRNAs participate in transcriptional regulation [29]. Our nucleocytoplasmic separation and RNA-FISH assays confirmed *circARID1A*’s cytoplasmic localization, providing spatial support for its miRNA sponge role in regulating *miR-147a*. Additionally, RNase R resistance validated its circular structure and stability [30], ensuring sustained regulatory activity. These data complement prior findings that *circARID1A* promotes GC via RNA-protein complexes [17], reinforcing the rationality of the *circARID1A*/*miR-147a*/SNAI2 axis and strengthening our mechanistic claims. Notably, the circular nature of *circARID1A* was further confirmed by Sanger sequencing of its back-splice junction, which matched the sequence previously reported in gastric cancer [17]. This cross-validation eliminates ambiguity between *circARID1A* and its linear host gene isoform, as standard qPCR alone cannot distinguish the two. If additional experimental evidence for the back-splice junction is required, we are willing to provide the original Sanger sequencing electropherogram in the revised version.

Earlier studies have reported that *circARID1A* is highly expressed in neuroblastoma, promoting cancer cell proliferation and metastasis by regulating related miRNAs and signaling pathways [16,31]. For instance, Fuchs et al. [16] identified circARID1A as one of the 25 circRNAs specifically upregulated in neuroblastoma, revealing its potential role in promoting malignancy through MYCN suppression. Similarly, Li et al. [31] found that interfering with *circARID1A* slows glioblastoma invasion by regulating the miR-370-3p/TGFBR2 pathway, demonstrating the impact of *circARID1A* on cancer cell migration and invasion. However, there are fewer studies on *circARID1A* in GC. Previous studies on *circARID1A* in gastric cancer are limited, with one notable study by Ma et al. [17] demonstrating that *circARID1A* interacts with IGF2BP3 and enhances tumor growth by assembling a *circARID1A*-IGF2BP3-SLC7A5 RNA-protein complex. The study further revealed that *circARID1A* is elevated in GC tissues and shows a positive correlation with tumor volume, tumor length, and TNM stage. Serving as a scaffold, *CircARID1A* enhances the interaction between IGF2BP3 and SLC7A5 mRNA, which in turn stabilizes SLC7A5 mRNA and drives GC proliferation through the AKT/mTOR pathway. Our findings complement this by revealing that circARID1A promotes GC cell proliferation and invasion via the *miR-147a*/SNAI2 signaling axis. *In vivo*, knockdown of *circARID1A* suppressed tumor growth in mice, further confirming its tumor-promoting role in GC. These results align with earlier studies in other cancer types, indicating a shared mechanism in which *circARID1A* modulates miRNAs and downstream signaling pathways to promote malignancy. However, unlike the MYCN suppression pathway in neuroblastoma and the *miR-370-3p*/TGFBR2 pathway in glioblastoma, our study highlights the specific involvement of the *miR-147a*/SNAI2 axis in GC. This difference underscores the potential tissue-specific roles of *circARID1A* and suggests that targeted therapies might need to be tailored according to the specific miRNA and signaling pathways involved in different cancer types. Our findings complement and extend this work by identifying a distinct regulatory mechanism, in which *circARID1A* promotes GC cell proliferation and invasion via the *miR-147a*/SNAI2 signaling axis. These two mechanisms may represent parallel and potentially cell context–dependent pathways through which *circARID1A* exerts its oncogenic effects in gastric cancer, reflecting the functional versatility of circRNAs in different molecular environments.

*MiR-147a* has been demonstrated to have tumor-suppressive effects in several cancers. It can enhance radiosensitivity of non-small cell lung cancer cells via suppressing STAT3 [32]. Many studies have found that *miR-147a* exhibits tumor-suppressive effects in multiple cancers by inhibiting cell proliferation and migration, a process often regulated by different circRNAs or lncRNAs [24,32]. Ding et al. [23] discovered that the progression of epithelial ovarian cancer was accelerated by circ_0072995 through modulation of the *miR-147a*/CDK6 axis. Additionally, Li et al. [33] showed that the LINC00847 accelerates non-small cell lung cancer progression by means of the E2F1-induced *miR-147a*/IFITM1 axis. In our study, *miR-147a* was found to be significantly downregulated in gastric cancer tissues and cells. Overexpression of *miR-147a* markedly inhibited tumor growth in nude mice. This study is the first to report the downregulation of *miR-147a* in GC and reveal its negative regulation by *circARID1A*. Results from the experiments demonstrate that overexpressing *miR-147a* markedly inhibits GC cell growth, migration, and invasion, further confirming its tumor-suppressive function.

*SNAI2* is a known transcription factor with oncogenic effects in various cancers, promoting migration and invasion through the EMT process. It is targeted and regulated by multiple miRNAs, playing a critical role in EMT and cancer invasion. By targeting *SNAI2*, *miR-203* effectively reduces the invasion and migration of breast cancer cells, and *miR-124* similarly inhibits their proliferation and migration through this target [34,35]. In gastric cancer, *miR-33a* inhibits EMT and related cell invasion and migration by targeting *SNAI2* [27]. This widespread regulation of *SNAI2* by miRNAs underscores its crucial role in cancer progression and highlights the precise control of its activity by various miRNAs. In GC, the high expression of *SNAI2* is closely associated with cancer invasiveness and metastasis [28,36]. This study showed that *miR-147a* can inhibit *SNAI2* expression by directly targeting the 3’UTR region of *SNAI2*, thereby suppressing the malignant behavior of GC cells. In this study, *miR-147a* was shown to inhibit *SNAI2* expression, and overexpression of *miR-147a* both in vitro and in vivo suppressed tumor growth, confirming the importance of *miR-147a*/SNAI2 regulation in GC progression. More importantly, *circARID1A* negatively regulates *miR-147a*, thereby indirectly upregulating *SNAI2* expression, which ultimately promotes the proliferation, migration, and invasion of GC cells, as demonstrated by both *in vitro* and *in vivo* experiments.

Despite these findings, several limitations should be acknowledged. Due to the lack of clinical ethical approval, paired gastric cancer and adjacent normal tissues were not available, and the clinical relevance of *circARID1A*, *miR-147a*, and *SNAI2* requires further validation in future studies. In addition, only subcutaneous xenograft models were used, which may not fully recapitulate the native gastric tumor microenvironment, and more clinically relevant orthotopic models should be considered. Notably, our in vivo findings are based on subcutaneous xenograft models, which primarily evaluate tumor growth rather than invasion or metastasis. Although in vitro transwell assays confirmed that *circARID1A* promotes GC cell invasive capacity, direct evidence of in vivo metastasis requires validation using tail vein injection lung metastasis models or orthotopic implantation models – this will be addressed in future studies. Moreover, although *circARID1A* was upregulated in GC, its upstream regulatory mechanisms remain unclear and warrant further investigation. Finally, although emerging evidence suggests that *circARID1A* may have prognostic value in gastric cancer [17], survival and treatment-response analyses could not be performed in the present study, and the use of the immortalized GES-1 cell line as a normal control should be interpreted with caution. Furthermore, key rescue experiments were validated in a second GC cell line (HGC-27), with results consistent with those in AGS cells. This confirms that the regulatory role of the *circARID1A*/*miR-147a*/SNAI2 axis is not cell-line specific, enhancing the robustness and generalizability of our findings.

In summary, this study systematically reveals for the first time the mechanism by which *circARID1A* promotes malignant proliferation in GC cells through the *miR-147a*/SNAI2 signaling axis, enriching our understanding of the molecular mechanisms of GC. The *in vivo* data further confirm the oncogenic role of *circARID1A* and the tumor-suppressive role of *miR-147a*, suggesting that the *circARID1A*/*miR-147a*/SNAI2 axis could serve as a novel target for therapeutic intervention. Nevertheless, this study has certain limitations. Although the study elucidates the critical role of the *circARID1A*/*miR-147a*/SNAI2 signaling axis in GC, the upstream regulatory mechanisms remain to be thoroughly investigated. Future research could explore the role of *circARID1A* in different subtypes of GC and its potential as a therapeutic target.

## Conclusions

This study reveals that *circARID1A* promotes GC cell proliferation, migration, and in vitro invasion by regulating the *miR-147a*/SNAI2 signaling axis. Knockdown of *circARID1A* inhibits these in vitro malignant behaviors and suppresses tumor growth in subcutaneous xenograft models by upregulating *miR-147a* and downregulating *SNAI2*. These findings highlight the potential of *circARID1A*, *miR-147a*, and *SNAI2* as therapeutic targets for GC.

## Supplementary Material

Supplemental Material

## Data Availability

The datasets used or analyzed during the current study are available from the corresponding author on reasonable request.
